# L-arginine attenuates Interleukin-1β (IL-1β) induced Nuclear Factor Kappa-Beta (NF-κB) activation in Caco-2 cells

**DOI:** 10.1371/journal.pone.0174441

**Published:** 2017-03-23

**Authors:** Qinghe Meng, Mitchell Cooney, Natesh Yepuri, Robert N. Cooney

**Affiliations:** Department of Surgery, SUNY Upstate Medical University, Syracuse, New York, United States of America; National Institute for Agronomic Research, FRANCE

## Abstract

**Background:**

Specific nutrients like L-arginine (L-Arg) ameliorate intestinal inflammation, however the exact mechanisms of this effect are unclear. We hypothesized the anti-inflammatory effects of L-Arg require active transport and metabolism by inducible nitric oxide synthase (iNOS) to generate nitric oxide (NO). To test this hypothesis we examined the effects of L-Arg, L-Arg transport activity, NO production and iNOS inhibitor on IL-1β-mediated NF-κB-activation in Caco-2 cells.

**Methods:**

Caco-2 cells were cultured, transfected with a NF-κB promoter luciferase vector, incubated ± L-Arg, ± IL-1β and luciferase activity was measured. Using siRNA we inhibited the L-Arg cationic amino acid transporter system y^+^ (CAT1) expression and examined its effects on L-Arg transport activity and IL-1β-mediated NF-κB-activation. Finally, the effects of sodium nitroprusside (SNP, a NO donor) and Nω-nitro-L-arginine (NNA, an iNOS inhibitor) on IL-1β-mediated NF-κB-activation were examined.

**Results:**

IL-1β increased NF-κB luciferase activity (8-fold) and NF-κB expression (mRNA and protein), both of these were significantly decreased by L-Arg. System y^+^ CAT1 siRNA decreased CAT1 expression, L-Arg transport activity and attenuated the inhibitory effects of L-Arg on NF- κB activity. SNP attenuated the IL-1β-induced increase in NF-κB luciferase activity and expression, whereas NNA diminished the inhibitory effects of L-Arg on IL-1β-inducible NF- κB luciferase activity.

**Conclusion:**

The inhibitory effects of L-Arg on IL-1β-mediated NF-κB-activation in Caco-2 cells involve L-Arg transport activity by CAT1, regulation of IL-1β-mediated increases in NF-κB expression, changes in iNOS expression and NO production. Our data suggest the inhibitory effects of L-Arg on NF-κB activation are mediated in part by iNOS since SNP preserves and NNA attenuates the effects of L-Arg on IL-1β-mediated NF-κB-activation and expression.

## Introduction

Maintenance of normal gastrointestinal physiology is essential for nutrient absorption, gut barrier function and host defense. Inflammatory bowel disease (IBD), necrotizing enterocolitis (NEC), sepsis and other clinically important diseases are characterized by intestinal inflammation and gut dysfunction [1; 2]. Consequently, identifying effective strategies to decrease intestinal inflammation and understanding their mode of action are critical to developing effective treatments for inflammatory intestinal conditions.

Several lines of evidence suggest enteral nutrition and conditionally essential amino acids like L-arginine (L-Arg) may be useful in treating certain types of intestinal inflammation [3]. L-Arg may be synthesized by intermediary metabolism of “precursor amino acids” like glutamine. However, during metabolic stress external supplementation of L-Arg may be necessary to meet the metabolic demands of stressed state [4]. Circulating levels of L-Arg are decreased in premature infants with NEC [5; 6]. Administration of intravenous L-Arg significantly attenuates NEC-induced intestinal damage in a neonatal porcine experimental model of NEC [7]. Furthermore, providing supplemental L-Arg to neonates at risk for NEC significantly reduces the risk of developing NEC [8].

Decreased intestinal inflammation has also been described following L-Arg administration in murine models of dextran sulfate sodium (DSS)-induced colitis, an experimental model of IBD [9; 10]. In DSS-colitis, L-Arg supplementation improves survival, weight loss, mucosal integrity and intestinal inflammation [9]. The beneficial effects of L-Arg administration in DSS-colitis are eliminated when studies are performed in inducible-nitric oxide synthase (iNOS) knockout mice suggesting an important role for iNOS and nitric oxide (NO) in attenuating gut injury.

L-Arg is actively transported into cells by the cationic amino acid transporter (CAT) family of transporter proteins [8; 11]. Although four CAT proteins have been described, CAT1 is constitutively expressed and is thought to be the major L-Arg transport protein in enterocytes [8; 11]. Once inside the cell there are several potential biochemical pathways for L-Arg metabolism: degradation by arginase to ornithine and urea, modification by L-Arg decarboxylase or glycine amidinotransferase or conversion to NO by nitric oxide synthase (NOS). Constitutive NOS is present at low levels in neuronal and endothelial tissues and enzyme activity is regulated by calcium [12]. In contrast, inducible NOS expression is minimal in resting cells and expression is markedly increased by inflammatory stimuli e.g. lipopolysaccharide (LPS), tumor necrosis factor (TNF) and interleukin 1 (IL-1) [13].

NO is a gaseous, highly reactive signaling molecule with a short half-life involved in regulating multiple physiological and pathophysiological processes including: vasodilation, phagocytic immune responses and reperfusion injury. Paradoxically, NO may have destructive and/or protective effects on tissue injury during inflammation [13].

The current study examines the anti-inflammatory effects of L-Arg on enterocytes using an *in vitro* model of intestinal inflammation. Caco-2 cells are derived from a human colon adenocarcinoma and are used extensively to characterize enterocyte physiology. Under the cell culture conditions utilized in this study, Caco-2 cells grown in monolayers demonstrate the same morphology and function as mature, well differentiated brush border enterocytes [14]. Interleukin 1β (IL-1β) is implicated in the pathogenesis of intestinal inflammation and stimulates nuclear factor- κB (NF-κB) activation which serves as an indicator of cellular inflammation [15; 16]. The current study provides evidence that the inhibitory effects of L-Arg on NF-κB-activation in Caco-2 cells involves CAT1 mediated L-Arg transport activity and are mediated in part by iNOS since the NO donor SNP preserves and iNOS inhibitor NNA attenuate the effects of L-Arg on IL-1β-mediated NF-κB-activation and expression.

## Materials and methods

### Reagents and plasmids

Recombinant human IL-1β was obtained from R&D Systems (Minneapolis, MN). L-arginine, sodium nitroprusside (SNP) and Nω-nitro-L-arginine (NNA) were obtained from Sigma Chemical Co. (St. Louis, MO). iκB-α wild-type expression vector, as well as the pNF-κB-Luc expression vectors (pCMV4) were kindly gifted by Dr. Shao-Cong Sun (M.D. Anderson, Houston, TX). DNA sequencing was performed to verify identities for all constructs before use. Three siRNA duplexes designed to target different coding regions of the human CAT1 mRNA sequence (Gene Bank Accession NO.NM_003045) were synthesized by Invitrogen (Carlsbad, CA). siRNA1 (5’- GCA GTG ATC ATA ATT CTC ATC TTG A -3’), siRNA2 (5’- CAG GAT TTG TGA AAG GAT CGG TTA A -3’), and siRNA3 (5’- CGC CAG TCT TCT AGG TTC CAT GTT T -3’). Three negative controls, 5’- GCA TAG TAC TAA TCT TAC TCG TTG A -3’ for siRNA1, 5’-CAG GTT TAA GTA GGA GGC TTA GTA A-3’ for siRNA2 and 5’- CGC TCT GAT CTT TGG TAC CGA CTT T-3’ for siRNA3 were also obtained from Invitrogen.

### Cell lines and growth conditions

Caco-2 cells were obtained from American Type Culture Collection (Rockville, MA) at Passage 27. Cells were maintained in stock cultures in Dulbecco’s Modified Eagle’s Medium (Gibco, Gaithersburg, MD) supplemented with 10% fetal bovine serum (Atlanta Biologicals, Flowery Branch, GA), 1% non-essential amino acids (100X, Gibco), 1 mM sodium pyruvate (Gibco), 2 mM L-glutamine (Gibco) and 1% antibiotic (Penicillin-Streptomycin, Gibco) in T-75 flasks at 37°C in a humidified incubator with 5% CO_2_. The stock Caco-2 cells were passaged every 5 days following treatment with 0.5% trypsin and 1 mM EDTA. Cells were re-seeded at a density of 5×10^6^ cells per T-75 flask for future sub-culturing, seeded into 6-well tissue culture plate at a density of 1.5×10^5^ cells per well for mRNA and protein assay, or a density of 1×10^4^ cells per well was grown in the tissue culture plate (24-well) for transport activity. At day 5, the cell reached to nearly 100% confluent, cells were utilized to measure transport activity. The growth media were refreshed every other days. A phase contrast microscope was used to monitor Caco-2 cells daily.

### Cell treatments

Cell monolayers were rinsed three times with PBS prior to treatments and re-incubated in serum-free media with 0.4 mM of L-arginine (Dulbecco’s Modified Eagle Medium supplemented with penicillin and streptomycin and without FBS, glutamine and sodium pyruvate) containing IL-1β (0 to 10 ng/ml) for different periods of time (0–6 hours). L-Arg (0 to 10 mM), SNP (NO donor: 0 to 10 mM) or NNA (NOS inhibitor: 0 to 1 mM) for different periods of time (0–6 hours) were used to treat cells in the presence or absence of IL-1β.

### Measurement of IL-6 levels and NO production

Enzyme-linked immunosorbent assay (ELISA) was used to determine cytokine IL-6 levels from cell culture media. Commercially available ELISA kits were used according to the manufacturer's instructions (R&D Systems Inc). NO production was assayed by using total nitric oxide assay kit (EMSNOTOT, Thermo Scientific, Frederick, MD) following the manufacturer's instructions. All samples were normalized to protein concentration and were assayed in triplicate.

### L-Arg transport activity

Caco-2 cell seeded in 24-well plates were administrated with the treatments. Each plate for transport contains both treatment and control groups. Transport assay were performed at 37°C ± 1°C. First cells were washed with three times with "transport buffer" (37°C) including, 1.2 mM MgSO_4_, 10 mM HEPES/Tris buffer (pH 7.4), 4.7 mM KCl, 2.5 mM CaCl_2_, 1.2 mM KH_2_PO_4_, and 10 mM leucine, 137 mM choline chloride. Then transport activity measurement was performed by applying 1 ml “transport buffer” supplemented with L-[3H] Arg (2 millicurie/ml). Tissue culture plates were placed at an incubator with an orbital shaking during the incubation process. L-Arg uptake was stopped by removing the “transport buffer” from each well with cells. Ice-cold “transport buffer” without substrate was applied to rinse cells for three times. The extracts from cells were obtained by using 1 ml 1N NaOH and then neutralized with acetic acid. Liquid scintillation spectrometry was used to measure isotope radioactivity. Protein concentrations from NaOH extract was assessed by Bio-Rad protein assay. Preliminary study showed that L-Arg transport was linear at 2 minutes under these assay conditions. Therefore the 2-minutes time point was chosen for transport experiments with zero time point serving as blank. System y^+^ L-Arg transport activity was transport activity which was not blocked by leucine and measured in choline chloride uptake buffer. Transport rate is expressed as nmoles of L-Arg per minute per milligram of cell protein concentration.

### Transfections of NF-κB and CAT1 siRNAs *in vitro*

Transfection plasmids were purified using HiSpeed Plasmid Maxi Kit (QIAGEN, Valencia, CA). siRNAs were obtained from Invitrogen. Caco-2 cells were seeded in 6-well plates with 60–80% confluence. Transfections were carried out using the either Effectene Transfection Reagent (QIAGEN Valencia, CA) or Lipofectin 2000 (Invitrogen). Transfection efficiency and stability in Caco-2 cells was evaluated using a plasmid which expresses green fluorescent protein (GFP) gifted by Dr. Gary Clawson (Department of Pathology, Penn State College of Medicine, Hershey, PA). The conditions of transfection in all promoter vectors and siRNAs were optimized (plasmid or siRNAs concentrations, transfection reagent concentration over time, etc.) in the beginning of experiments. Cells were transfected with NF-κB expression vector or co-transfected with iκB-α wild-type into the cells when appropriate using Effectene Transfection Reagent. Empty vector (pCMV4) was utilized to be a control in all experiments. Similarly, CAT1 siRNAs or negative control were transfected with Lipofectin 2000 as directed by the manufacturer’s protocol. Transfections were allowed to proceed for 20–24 h before the reagent mixture was removed. Then cells were washed with PBS before adding fresh medium.

### Luciferase activity and protein assays

20 μl of cell extracts were employed for measuring luciferase activity by using Luciferase Assay System (Promega, Madison, WI) and a Berthold SIRIUS Luminometer (Oak Ridge, TN). First cells were co-transfected with a β-galactosidase expression vector, and then luciferase and β-galactosidase activity were measured. Initially luciferase activity was normalized to β-galactosidase activity and expressed as relative light units. Meanwhile, 2 μl of cell extracts were used for total protein concentration by Bio-Rad assay. Luciferase activity normalized to total protein from cell extracts produced similar results. In final results, the luciferase activity normalized to protein is reported as fold induction (fold change) compared to the time-matched control group in all experiments.

### Measurement of NF-κB protein level

Equal amounts of protein (20 μg) were separated by SDS-PAGE (Pierce, Rockford, IL) and transferred to polyvinylidene difluoride membranes (Millipore, Bedford, MA). Blots were blocked for 1 h at room temperature in TBS-T (150 mM NaCl, 10 mM Tris, pH 8.0, 0.1% Tween 20) containing 5% nonfat dry milk. Blots were incubated with polyclonal antibodies (sc-109, Santa Cruz, CA) in TBS-T containing 5% nonfat dry milk overnight at 4°C. The blots were incubated with a horseradish peroxidase-conjugated (HRP) goat anti-rabbit antibody (Rockland, Gilbertsville, PA) for 1 h at room temperature after several washes with TBS-T. Then blots were washed in TBS-T and proteins were visualized by exposure to Luminol Enhancer Solution (Thermo Scientific Rockford, IL) according to the manufacturer’s instructions. The blots were stripped by incubation with Restore Western Blot stripping Buffer (Thermo Scientific Rockford, IL) at room temperature for 10 min. β-Actin (Santa Cruz, CA) was used to verify equal protein loading. Quantification of band intensity was obtained by using a calibrated densitometer with Quantity One software (GS800, Bio-Rad, Hercules, CA). Immunoblot results are expressed as relative densitometry units (RDU) normalized to β-Actin.

### Measurement of NF-κB and iNOS mRNA levels

Total RNA was isolated from cells by the TRIzol method (GIBCO BRL, Life Technologies). Total RNA (2 μg) was converted to cDNA using an iScript cDNA Synthesis Kit (Bio-Rad Laboratories, Inc. Hercules, CA). PCR was performed with the Step One Plus Real-Time PCR System with Step One software V2.0 (Applied Biosystems, Forster City, CA) according to the manufacturer’s guidelines. qRT-PCR was performed using iQ SYBR Green Mix (Bio-Rad, Hercules, CA) according to the manufacturer’s protocol. Relative gene expression was determined by the CT method, and mRNA levels were normalized to β-Actin. For PCR amplification, the specific primers used were NF-κB (Sense 5’- CAT GGC TGA AGG AAA CCA GTG CAA -3’ and antisense 5’- AGA GCA AGG AAG TCC CAG ACC AAA -3’), iNOS (Sense 5’- TCC GAG GCA AAC AGC ACA TTC A -3’ and antisense 5’- GGG TTG GGG GTG TGG TGA TGT -3’), β-Actin (Sense 5’- AGC GGG AAA TCG TGC GTG AC -3’ and antisense 5’- TCC ATG CCC AGG AAG GAA GG -3’).

### Measurement of system y^+^ CAT1 mRNA level

Northern blot analysis was used to examine system Y^+^ CAT1 mRNA after total RNA was isolated from Caco-2 cells. RNA was separated on a 1% formaldehyde gel, transferred to nylon membrane (Genescreen, New England Nuclear), The membrane was hybridized with an antisense oligonucleotide probe specific to rat CAT1 (5’- GTA GAA GTG GCC TAG CTC CTC G-3’) and then stripped and rehybridized with an oligonucleotide probe specific for β-actin RNA (5’- ATT TCC CGC TCG GCC GTG GTG GTG AAG CTG TAG C -3’). System y^**+**^ CAT1 signal was quantified by using laser densitometer to scan autoradiographs and normalized to β-actin RNA. The 3’ end-labeled system y^**+**^ CAT1 probe was made by using terminal transferase and α-[^32^P] dATP. 5’ end-labeled β-actin probe was made by using T4 polynucleotide kinase and ^32^P-ATP.

### Statistical methods

Data are presented as means ± SEM. The number of samples in each group is specified in the figure legends. Statistical analysis is performed using Student’s t-test or ANOVA followed by Student-Newman-Keuls post testing using Prism 4.0 (GraphPad Software, San Diego, CA). Differences among groups were considered significant at *P≤* 0.05.

## Results

### Effect of L-L-Arg on NF-κB activation, expression and IL-6 production by IL-1β

**Fig 1:**
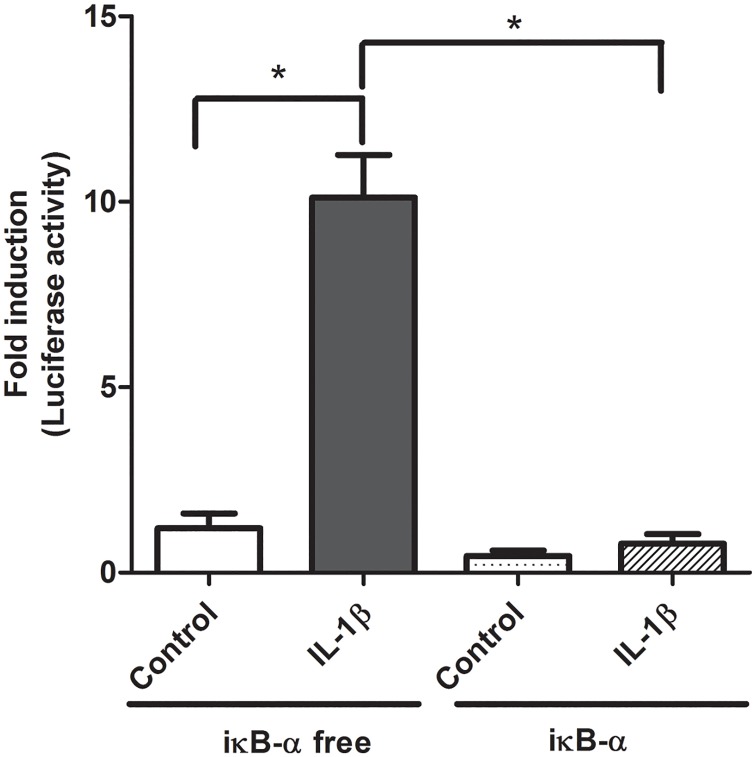
Effects of iκB-α and IL-1β-inducible NF-κB activation. Caco-2 cells were transfected with pNF-κB-Luc for 18 hours, then treated±IL-1β (4 ng/ml) for 4 hours. Cell lysates were assayed for luciferase activity and protein concentration as described in *Methods*. Data are expressed as fold induction of normalized luciferase activity ± SE. * *P*<0.05 (n = 6 /group).

**Fig 2:**
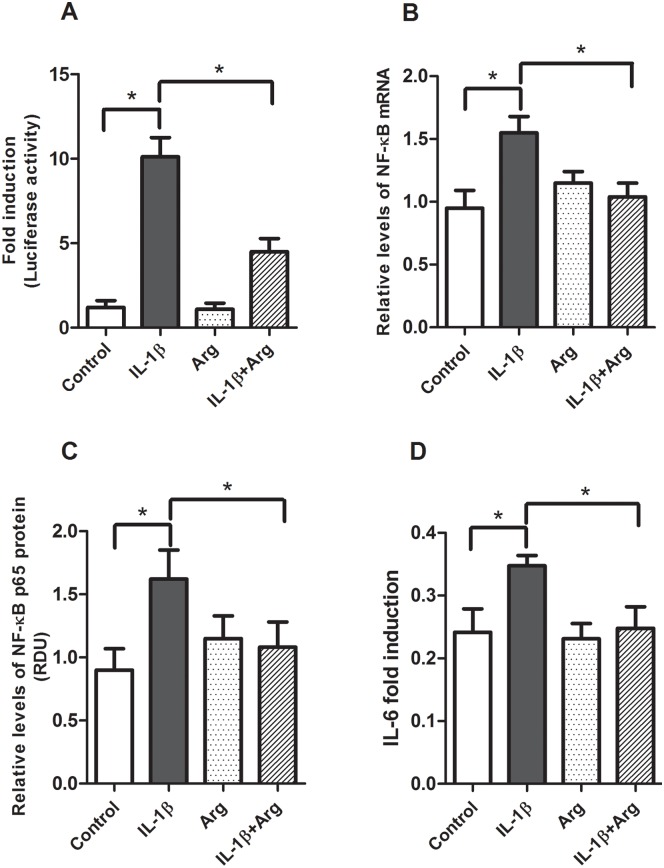
Effects of L-Argon IL-1β-induced NF-κB promoter activity, expression and IL-6 production. Caco-2 cells transfected with pNF-κB-Luc vector were treated with or without IL-1β (4 ng/ml) for 4 hours after pretreatment in the absence or presence of L-Arg (5 mM) for 4 hours (Fresh medium with L-Arg was applied after removing medium for pre-treatment). Cells were harvested for luciferase activity, isolations of total RNA and protein. A: NF-κB luciferase activity data are expressed as fold induction of normalized luciferase activity. B: NF-κB mRNA levels were determined by qRT-PCR, then normalized to β-actin. C: NF-κB p65 protein levels were measured by Western blot analysis and normalized to β-actin. D: IL-6 levels in the cell culture media were measured by ELISA, normalized to total protein and expressed as fold-induction. All data are means± SE, * *P*<0.05. (n = 5–6 /group).

We began by establishing our cell culture model of intestinal inflammation. Caco-2 cells transfected with the pNF-κB-Luc vector and stimulated with IL-1β (4 ng/ml) demonstrated an 8-fold increase in NF-κB promoter activity (Fig 1). Co-transfection with a wild-type iκB-α expression vector serves as a positive control to demonstrate our experimental model is responsive to inhibitors of NF-κB activation (Fig 1). Next, we examined the ability of L-Arg to inhibit NF-κB activation and expression. IL-1β significantly increased NF-κB activation, NF-κB mRNA (1.4 fold, Fig 2B) and protein expression (1.5 fold, Fig 2C). When Caco-2 cells were transfected with pNF-κB-Luc and incubated with L-Arg (5 mM) for 4 hours prior to treatment with IL-1β, NF-κB promoter activity was markedly decreased (Fig 2A). In addition, incubation with L-Arg attenuated the IL-1β mediated increase in NF-κB p65 mRNA (Fig 2B) and protein (Fig 2C) expression. To validate the role of L-Arg in modulating intestinal inflammation, we examined the effects of IL-1β and L-Arg on IL-6 production. As shown in Fig 2D, the IL-1β-induced increase in IL-6 was attenuated by L-Arg supplementation.

### Regulation of IL-1β mediated inflammation by L-Arg transport activity

**Fig 3:**
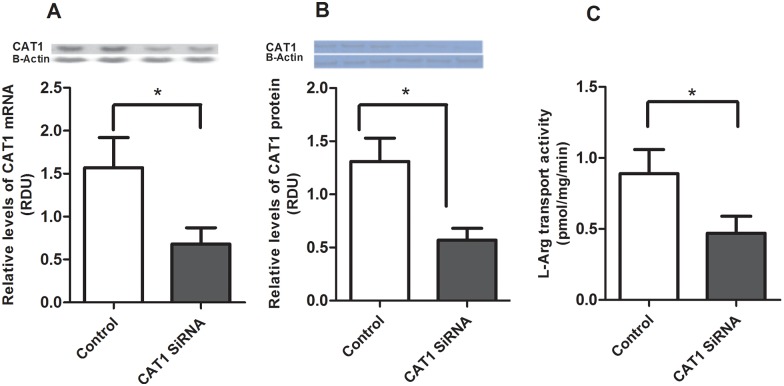
System y^+^ CAT1 siRNA knocked down L-Arg transport activity, CAT1 mRNA and System y^+^ protein. Caco-2 cells were transfected system y^+^ CAT1 siRNA for 24 hour as according to manufacturer’s instruction. L-Arg transport activity was measured as described in *Methods*. Cell lysates were assayed for CAT1 mRNA and protein. A: CAT1 mRNA. The levels of CAT1 mRNA were determined by Northern Blot analysis, then normalized to β-actin. System y^+^ CAT1 siRNA knocked down CAT mRNA by 60%. B: System y^+^ protein. Western blot analysis was used to detect System y^+^ protein and normalized to β-actin. C: L-Arg transport activity. Transport activity was expressed as picomoles L-Arg per milligram protein per 1 min of uptake. Data are means ± SE, * *P*<0.05. (n = 6 /group).

**Fig 4:**
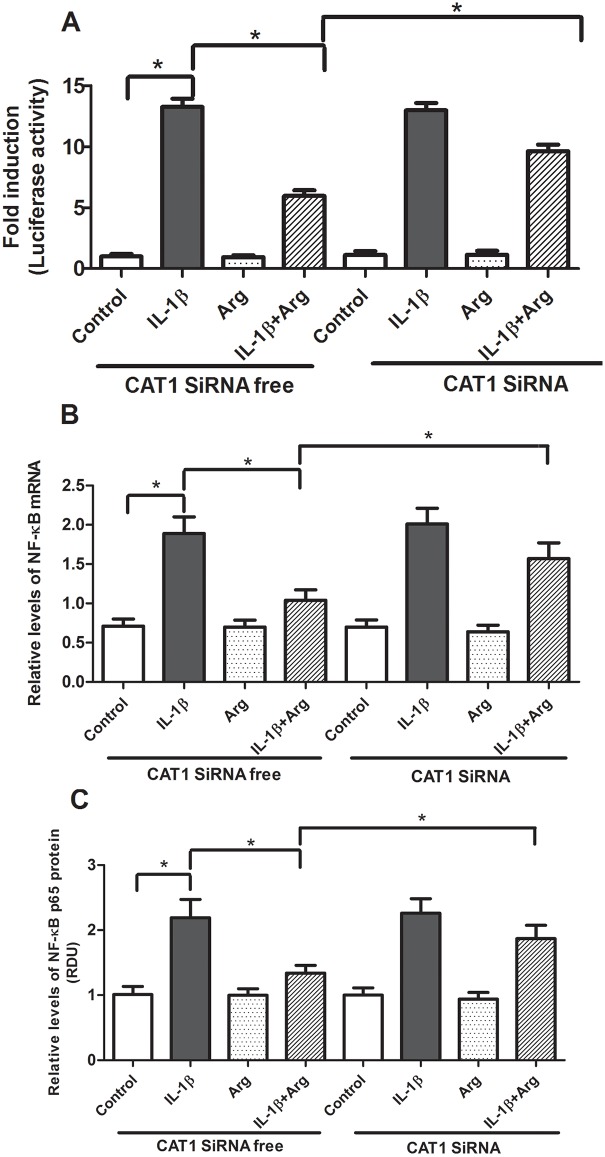
Effects of system y^+^ CAT1 siRNA and L-Arg’s ability on NF-κB promoter activity and NF-κB expression. Caco-2 transfected with system y^+^ CAT1 siRNA for 24 hour prior to the transfection of pNF-κB Luc vector for 18 hours were treated with IL-1β (4ng/ml) for 4 hours after pretreatment of 4 hours of L-Arg (5 mM) (Fresh medium with L-Arg was applied after removing medium for pre-treatment). Cells were collected for luciferase activity and isolations of protein and total RNA as described in *Methods*. A: NF-κB luciferase activity. Data are expressed as fold-induction of normalized luciferase activity. B: NF-κB mRNA levels were determined by qRT-PCR, then normalized to β-actin. C: NF-κB p65 protein levels were measured by Western blot analysis and normalized to β-actin. Data are means ± SE. * *P*<0.05 (n = 6 /group).

System y^+^ CAT1 is responsible for transporting L-Arg and accounts for approximately 70% of L-Arg transport ^17^. To determine whether CAT1 mediated L-Arg transport is required for the inhibitory effects of L-Arg on IL-1β mediated inflammation, we transfected Caco-2 cells with CAT1 siRNA for 24 hours. Transfection with CAT1 siRNA decreased the relative abundance of CAT1 mRNA (Fig 3A) and protein (Fig 3B) by more than 50% and significantly reduced L-Arg transport activity (Fig 3C) in Caco-2 cells. These data confirm system y^+^ CAT1 is an important L-Arg transport in Caco-2 cells. Next, Caco-2 cells were transfected with CAT1 siRNA or control siRNA for 24 hours, then transfected with pNF-κB Luc vector for 18 hours and treated ± L-Arg and ± IL-1β. As shown in Fig 4, the inhibitory effects of L-Arg on IL-1β-mediated NF-κB activation and expression are diminished by transfection of CAT1 siRNA.

### Regulation of IL-1β mediated inflammation by SNP and iNOS

**Fig 5:**
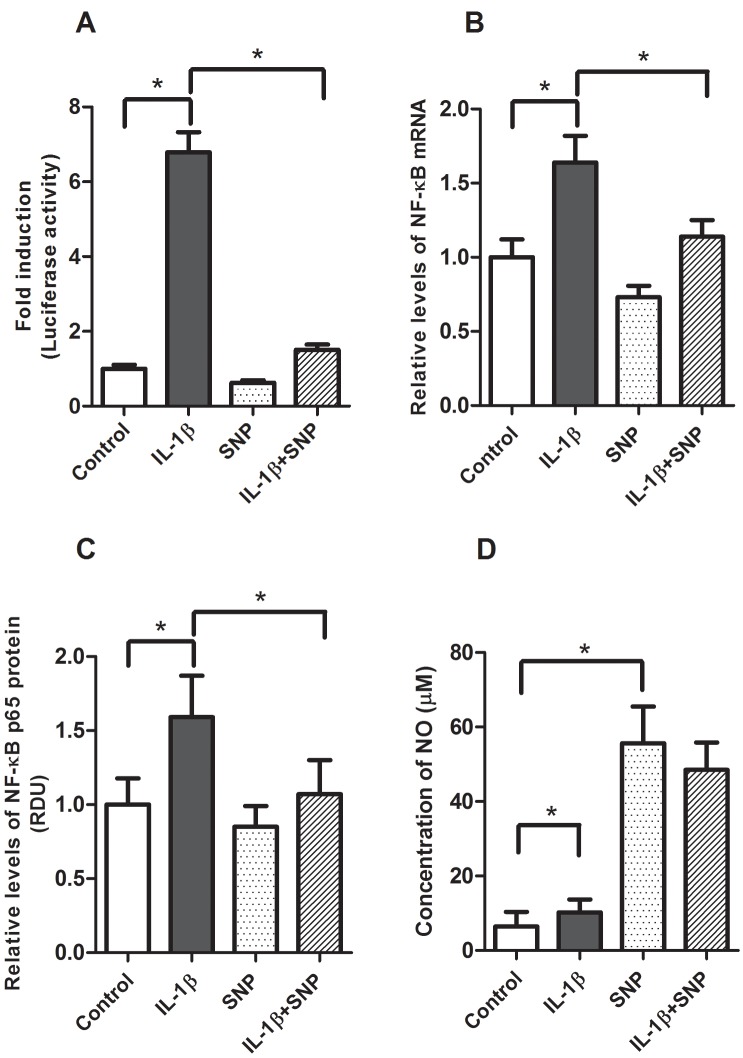
Effects of SNP in IL-1β inducible NF-κB promoter activity, expression and NO production. Caco-2 cells were transfected with pNF-κB-Luc vector were treated with IL-1β (4 ng/ml) for 4 hours after pretreatment of SNP (1 mM) for 4 hours. Cells were harvested for luciferase activity and isolations of protein and total RNA. A: NF-κB luciferase activity, data are expressed as fold-induction of normalized luciferase activity. B: NF-κB mRNA levels were determined by qRT-PCR, then normalized to β-actin. C: NF-κB p65 protein was measured by Western blot analysis and normalized to β-actin. D: NO levels in cell culture media were measured as described in Methods. Data are means ± SE, * *P*<0.05. (n = 4–6 /group).

A potential mechanism for the inhibitory effects of L-Arg on IL-1β mediated inflammation is metabolism by iNOS to generate NO. To investigate this possibility, we examined the effects of SNP, a nitric oxide donor, on IL-1β-mediated inflammation and NO production. Caco-2 cells transfected with pNF-κB-Luc vector were pretreated with SNP (1 mM) for 4 hours, then stimulated with IL-1β. As shown in Fig 5, SNP significantly decreased IL-1β-mediated NF-κB activation (Fig 5A), NF-κB mRNA (Fig 5B) and protein expression (Fig 5C). As shown in Fig 5D, IL-1β caused a slight increase in NO concentration, whereas media containing SNP demonstrated a more than 5-fold increase in NO levels.

**Fig 6:**
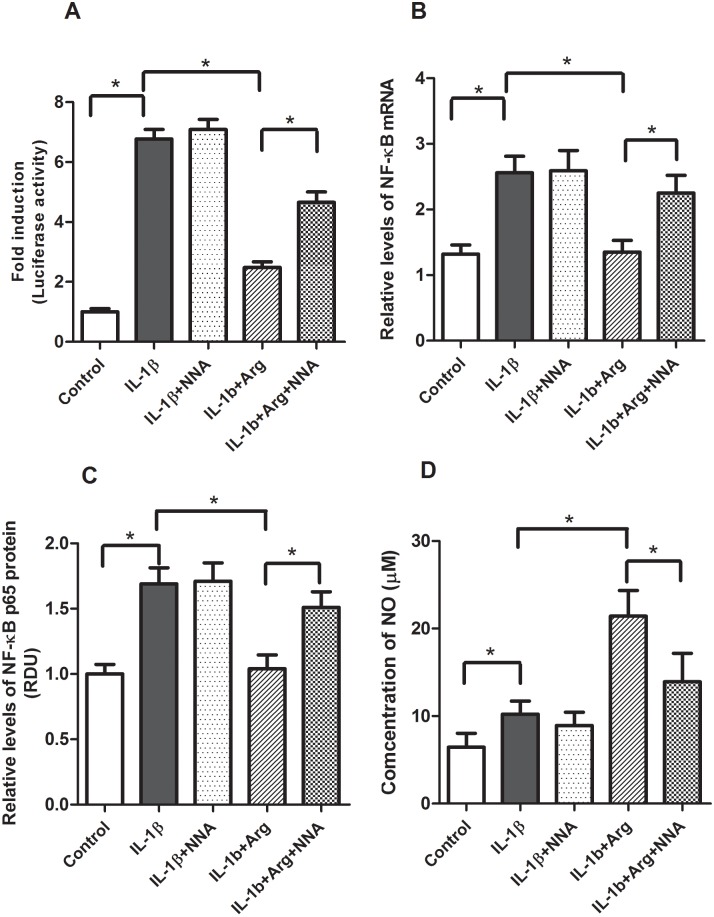
Effects of *Nω*-Nitro-L-Arginine (NNA) on IL-1β inducible NF-κB promoter activity, expression and NO production. Caco-2 cells transfected with pNF-κB-Luc vector were treated with IL-1β (4 ng/ml) for 4 hours after pretreatment of NNA (0.5 mM) and L-Arg (5 mM) for 4 hours (Fresh medium with NNA and L-Arg was applied after removing medium for pre-treatment). Cells were harvested for luciferase activity and isolations of protein and total RNA. A: NF-κB luciferase activity is expressed as fold-induction of normalized luciferase activity. B: NF-κB mRNA were measured by qRT-PCR, then normalized to β-actin. C: NF-κB p65 protein was measured by Western blot analysis normalized to β-actin. D: NO levels in cell culture media were measured as described in Methods. NNA alone did not affect the LPS-induced NF-κB promoter activity, NF-κB mRNA and protein expression levels (not shown). Data are means ± SE, * *P*<0.05. (n = 4–6 /group).

Next we investigated the effects of NNA, an inhibitor of iNOS, on L-Arg’s ability to regulate IL-1β-mediated inflammation. Caco-2 cells were transfected with pNF-κB-Luc in the presence or absence of L-Arg and NNA (0.5 mM). In these experiments (Fig 6), NNA had no effect on IL-1β-mediated NF-κB activation, expression or NO levels. However, NNA attenuated the inhibitory effects of L-Arg on IL-1β-mediated NF-κB promoter activation (Fig 6A), NF-κB mRNA (Fig 6B) and NF-κB p65 protein (Fig 6C) expression. NNA also attenuated the increase in NO observed in the IL-1β+L-Arg treated cells (Fig 6D). Collectively these data suggest the inhibitory effects of L-Arg on IL-1β mediated inflammation in Caco-2 cells are mediated in part by cellular uptake metabolism of L-Arg to NO by iNOS.

**Fig 7:**
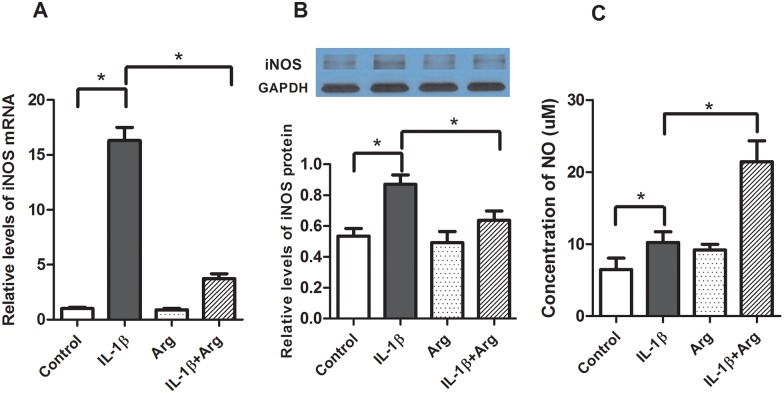
Effects of L-Arg and IL-1β on iNOS expression. Caco-2 cells were treated with IL-1β (4 ng/ml) for 4 hours after an incubation of L-Arg (5 mM) for 4 hours (Fresh medium with L-Arg was applied after removing medium). Cells were harvested for the isolation of total RNA and protein. A: iNOS mRNA was determined by qRT-PCR and normalized to β-actin. B: iNOS protein was measured by Western blot analysis normalized to β-actin C: NO levels in cell culture media were measured as described in Methods. Data are means ± SE, * *P*<0.05. (n = 6 /group).

To further investigate the mechanisms by which IL-1β and L-Arg regulate intestinal inflammation we examined their effects on iNOS expression (mRNA and protein) and activity (NO production) in Caco-2 cells. As shown in Fig 7, stimulation with IL-1β causes a more than 10-fold increase in iNOS mRNA levels, 1.6 fold increase in iNOS protein, but only a slight increase in NO. Treatment with L-Arg alone had essentially no impact on iNOS expression or NO levels in media (Fig 7). In contrast, while L-Arg treatment significantly attenuates the IL-1β-mediated increase in iNOS expression, it results in a marked increase in NO production.

## Discussion

The pathogenesis of intestinal inflammation is complex and involves alterations in gut barrier function at the host-microbe interface resulting in activation of the innate immune system. However several lines of evidence suggest NF-κB activation in mucosal epithelia represents a critical event in this process. Anti-inflammatory therapies like antibodies to TNF and steroids which regulate NF-κB activation are commonly used to treat intestinal inflammation in IBD, but are associated with significant side effects. Although elemental diets and specific nutrients like glutamine and L-Arg have been shown to attenuate gut inflammation, it is unclear whether they act by altering the microbiome, innate immunity and/or the cellular response to inflammation.

The current study provides a detailed investigation on the regulation of IL-1β mediated NF-κB activation by L-Arg in Caco-2 intestinal epithelial cells. While the Caco-2 cell line is originally derived from human colon adenocarcinoma, after two to three weeks of appropriate culture conditions the cells differentiate into a monolayer of polarized cells which expresses many morphological and functional features of small intestine enterocytes and are commonly used to study intestinal epithelial biology [17; 18].

The NF-κB family of transcriptional proteins includes: RelA (p65), RelB, C-Rel, NFκB1 (p50) and NFκB2 which associate to form transcriptionally active hetero- and homo-dimeric complexes [18]. The p65/p50 heterodimer is the most common and well characterized form of NF-κB. Normally, the p65 and p50 proteins are sequestered in the cytoplasm in an “inactive form” in a complex with the iκB protein [19]. Upon activation by inflammatory cytokines (TNF or IL-1), degradation of the iκB proteins allows p65 and p50 to dissociate, form dimers and translocate to the nucleus where they stimulate inflammatory gene expression by binding to NF-κB-binding elements in the promoter region of “target genes” [18]. While multiple stimuli (e.g. bacteria and their products) have been shown to activate NF-κB *in vivo* [20]. Our data showing activation of NF-κB promoter activity by IL-1β and its inhibition by iκB-α provides important positive and negative controls for subsequent experiments with L-Arg in our Caco-2 cell culture model [16].

L-Arg is a conditionally essential amino acid that serves to modulate immune responses. In mice, dietary L-Arg supplementation alters the gut microbiome resulting in a shift from Firmacutes to Bacteroides and down regulates jejunal NF-κB mRNA expression [12]. In patients with active Crohn’s disease, colonic biopsies cultured with glutamine and L-Arg demonstrate decreased TNF-α production and decreased expression of the NF-κB p65 protein [21]. Consistent with these observations, our data show incubation with L-Arg decreases IL-1β-induced NF-κB promoter activity, NF-κB mRNA and NF-κB P65 protein levels in Caco-2 cells. These findings provide evidence L-Arg regulates IL-1 mediated NF-κB activation and expression in cultured enterocytes.

To further characterize the mechanisms by which L-Arg regulates IL-1β mediated inflammation we examined the role of L-Arg transport. L-Arg is transported into cells via the y^+^ or cationic amino acid transport system (CAT). The CAT family includes four isoforms (CAT1, CAT2A, CAT2B, CAT3) which are expressed differentially in various tissues [22]. The CAT1 isoform is the predominant L-Arg transporter in intestinal epithelium and Caco-2 cells and has been shown to play a role in L-Arg-mediated elevations of NO via iNOS in intestinal epithelial cells [17; 23; 24]. L-Arg transport is increased 5-fold in brush border membrane vesicles from septic rats and in Caco-2 cells exposed to LPS and interferon-γ [23; 24; 25]. Previous work from our laboratory has shown LPS stimulates L-Arg transport activity by increase in CAT1 mRNA and protein levels of L-Arg transporter, and functional transporter units in IEC-6 cells [26]. The reductions in CAT1 mRNA, protein and L-Arg transport we observed following siRNA treatment of Caco-2 cells are consistent with previous studies identifying CAT1 as the principal L-Arg transporter in this cell line [17]. Furthermore, the suppression of IL-1β-induced NF-κB activity by L-Arg was significantly reduced by CAT1 siRNA. These results provide evidence CAT1 mediated L-Arg transport is important for the inhibitory effects of L-Arg on NF-κB activation.

As previously noted, L-Arg is the sole substrate for iNOS. There is considerable evidence iNOS and NO are important mediators of intestinal inflammation in diseases like IBD [27]. iNOS expression, activity and NO production are all increased in intestinal tissue from IBD patients. Although studies examining the effects of iNOS inhibition in intestinal inflammation have shown conflicting results, there is evidence the protective effects of L-Arg supplementation are mediated by NO production. NO has been shown to inhibit the activity of NF-κB by S-nitrosylation of the p50 subunit [28; 29]. In HCT-8 cells L-Arg supplementation was associated with increased NO production and decreased IL-8 during inflammation and the protective effects of L-Arg supplementation in DSS colitis are eliminated in iNOS knockout mice [9; 30]. The inhibitory effects of SNP on IL-1β mediated NF-κB activation/expression, the ability of the iNOS inhibitor NNA to prevent L-Arg’s down regulation of NF-κB activation/expression and the ability of L-Arg to attenuate the IL-1β mediated increase in iNOS mRNA and protein expressions are consistent with our hypothesis and the results of others [9; 30].

In summary, our findings show IL-1β stimulates NF-κB activation and expression which is attenuated by the addition of L-Arg in Caco-2 cells. The anti-inflammatory effects of L-Arg appear to involve active transport by CAT1 and metabolism by iNOS to generate NO. While these results improve our understanding of the mechanisms by which L-Arg regulates intestinal inflammation in a Caco-2 cell culture model, it is important to point out several limitations. First, while our data is derived from an established *in vitro* cell culture model, it may not be completely translatable to the human host and intestinal inflammation in all patients. Although the experiments were conducted with minimal media, defined exogenous L-Arg and without glutamine, we are unable to fully examine the effects of intermediary metabolism and endogenous L-Arg production. Finally, there are conflicting data in the literature on the clinical benefits of L-Arg supplementation in surgical patients and other experimental models [31; 32].
